# Structural basis for suramin binding to the C-terminal domain of the SARS-CoV-2 nucleocapsid protein: Structural basis of the interaction of SARS-CoV-2 N-CTD with suramin

**DOI:** 10.3724/abbs.2025182

**Published:** 2025-10-09

**Authors:** Chenyun Guo, Xiao Li, Hao Xu, Jiaxin Yu, Jia Li, Donghai Lin

**Affiliations:** Key Laboratory for Chemical Biology of Fujian Province College of Chemistry and Chemical Engineering Xiamen University Xiamen 361005 China

**Keywords:** NMR spectroscopy, nucleocapsid C-terminal domain (N-CTD), RNA-protein interaction, SARS-CoV-2, suramin

## Abstract

The global threat posed by COVID-19 persists, largely due to the high mutability of SARS-CoV-2 and the limited availability of effective antiviral therapeutics. The nucleocapsid (N) protein of SARS-CoV-2 is an attractive drug target because of its high degree of sequence conservation and essential role in viral replication. In this study, we show that suramin, a polysulfonated antiviral compound, binds to the C-terminal domain (N-CTD) of the N protein and interferes with its interaction with RNA. Biolayer interferometry (BLI) shows that suramin has a higher binding affinity for N-CTD ( *K*_d_, 3.30 μM) than for RNA ( *K*_d_, 10.12 μM). Electrophoretic mobility shift assays (EMSAs) further confirms that suramin effectively displaces RNA from N-CTD. NMR titration experiments and site-directed mutagenesis identify the α1-η1 helix (residues 248–262) as the primary suramin binding region, with residues K256, R259 and R262 playing critical roles in ligand recognition. In addition, NMR relaxation and model-free analyses reveal that the α1-η1 helix is highly flexible on the picosecond to nanosecond timescale, a dynamic feature that likely facilitates ligand binding. Furthermore, ITC and EMSA experiments demonstrate that suramin can bind to the full-length N protein at multiple sites and dissociate RNA from the N protein. Taken together, these findings provide structural and biophysical insights into the mechanism of action of suramin and establish a rational basis for the development of targeted antiviral therapies against SARS-CoV-2.

## Introduction

Remarkable progress has been made in understanding COVID-19 in terms of virology, treatment and prevention strategies. However, the high mutability of its causative agent, SARS-CoV-2, continues to present formidable challenges. SARS-CoV-2 harbors a large positive-sense RNA genome encoding four structural proteins—a spike (S), an envelope (E), a membrane (M), a nucleocapsid (N) and sixteen non-structural proteins (nsp1–16) [ 1 – 3]. Among these proteins, the N protein is highly conserved and plays a central role in the packaging and assembly of viral particles [4]. It binds to the viral RNA genome to form ribonucleoprotein (RNP) complexes, which are essential for efficient viral replication [5]. In addition, the N protein modulates host immune responses by interacting with innate immune signaling pathways [ 6, 7], making it a promising therapeutic target.

The full-length N protein consists of 419 amino acids and has two structured domains: the N-terminal domain (N-NTD, residues 44–180) and the C-terminal domain (N-CTD, residues 247–364), which are separated and flanked by intrinsically disordered regions (IDRs: residues 1–43, 181–246, and 365–419) [8]. Both N-NTD and N-CTD exhibit RNA-binding capabilities with enriched positively charged residues [ 9– 11]. In our previous investigation, we demonstrated that suramin, a polysulfonated naphthylurea compound, binds to the N-NTD of SARS-CoV-2 and disrupts its interaction with RNA [12]. Other studies have shown that suramin inhibits SARS-CoV-2 replication and reduces the viral load in human lung epithelial cells [13]. Although suramin has also been shown to inhibit key viral enzymes, such as RNA-dependent RNA polymerase (RdRp) and the 3CL protease [ 14, 15], its precise mechanism of action remains poorly defined.

Drug repurposing strategies, particularly those involving suramin, offer a rapid and efficient route to antiviral development. In this study, we show that the C-terminal domain of the SARS-CoV-2 N protein also binds to suramin and competitively displaces RNA. Our findings elucidate the structural basis of this interaction and provide a rational framework for the design of targeted antivirals against SARS-CoV-2.

## Materials and Methods

### Gene-fragment cloning and protein preparation

The pET28a plasmid encoding the full-length SARS-CoV-2 *N* gene was kindly provided by the Guangdong Provincial Institute of Laboratory Animal Monitoring (Guangzhou, China). The boundaries of the N-CTD were defined on the basis of the crystal structure (PDB ID: 6YUN). A cDNA fragment corresponding to residues 247–364 of the N-CTD was amplified and subcloned and inserted into the pSUMO expression vector (Novagen, Gibbstown, USA) via the *Bam*HI and *Nde*I restriction sites. Site-directed mutagenesis PCR was used to generate point mutants, and the resulting plasmids encoding both wild-type and mutant N-CTD constructs were transformed into *Escherichia coli* BL21(DE3) cells.

For protein expression, N-CTD was fused to an N-terminal hexahistidine (His _6_) tag followed by a SUMO tag (His-SUMO-N-CTD) and expressed in *E*. *coli* BL21(DE3) cells. Cultures were grown at 37°C in LB media supplemented with 50 μg/mL kanamycin (Sangon Biotech, Shanghai, China) until the optical density at 600 nm reached 0.6. Protein expression was induced with 0.5 mM isopropyl-β-D-1-thiogalactopyranoside (IPTG) and continued at 25°C for 10 h. For isotopic labelling, ^15^N-labelled N-CTD was expressed in M9 minimal medium supplemented with 0.1% (w/v) ^15^ NH _4_Cl as the sole nitrogen source. The cells were harvested via centrifugation and resuspended in buffer A (50 mM NaH _2_PO _4_, 50 mM Na _2_HPO _4_, and 300 mM NaCl, pH 6.5) containing 1 mM phenylmethylsulfonyl fluoride (PMSF). After cell lysis by sonication on ice and centrifugation, the supernatant was applied to a 5-mL Ni-NTA affinity column (GE Healthcare, Chicago, USA).

After removal of non-specifically bound proteins with imidazole gradients, the His-SUMO-N-CTD fusion protein was eluted with buffer A containing 500 mM imidazole, followed by digestion with SUMO-protease at 4°C overnight to remove the tag. The cleaved N-CTD was purified by a second round of Ni-affinity chromatography and eluted with buffer A containing 20 mM imidazole. Final purification was performed by size exclusion chromatography (SEC) on a Superdex 75 10/300 GL column (GE Healthcare) equilibrated with buffer B (25 mM Na _3_PO _4_ and 50 mM NaCl, pH 6.0) at a flow rate of 0.6 mL/min.

### Biolayer interferometry assays

Biolayer interferometry (BLI) experiments were performed using a ForteBio Octet96 instrument (ForteBio, Inc., San Francisco, USA). A 15-nucleotide RNA sequence (5′-AUAUGGAAGAGCCCUA-3′) derived from the SARS-CoV-2 genome was synthesized by GenScript (Nanjing, China). Suramin (product number S2671-100MG) was purchased from Sigma-Aldrich (St Louis, USA). Biotinylated wild-type and mutant N-CTD proteins were immobilized on super streptavidin (SSA) biosensors. All proteins and ligands were prepared in buffer B (25 mM Na _3_PO _4_ and 50 mM NaCl, pH 6.0).

For each assay, 20 μM biotinylated N-CTD was loaded onto the biosensor, followed by exposure to serial dilutions of RNA or suramin at concentrations of 0.39 μM, 0.78 μM, 1.56 μM, 3.12 μM, 6.25 μM, 12.5 μM, and 25.0 μM. After ligand binding, the biosensors were transferred to fresh buffer B for dissociation measurements. SSA biosensors without protein immobilization incubated with identical ligand concentrations served as negative controls. All binding curves were processed and analyzed using ForteBio Data Analysis 9.0 software to determine the equilibrium dissociation constant ( *K*_d_) for each protein-ligand interaction.

### Electrophoretic mobility shift assay

To evaluate the interaction between N-CTD and RNA, a series of binding reactions were prepared in buffer B. Each 20-μL reaction mixture contained 10 μM RNA and varying concentrations of N-CTD (10 μM, 20 μM, 40 μM, 60 μM, 80 μM, and 100 μM). The mixtures were incubated at room temperature (25°C) for 30 min and then resolved on a 20% native polyacrylamide gel. To evaluate the disruptive effect of suramin on N-CTD-RNA association, suramin was added to a fixed mixture of 30 μM N-CTD and 10 μM RNA at molar ratios of 1:1, 1:5 and 1:10 (N-CTD:suramin). These 20-μL samples were incubated as described above and resolved using the same gel conditions. After electrophoresis, nucleic acid bands were visualized by staining with 4S Red Plus Nucleic dye (Sangon Biotech) in a staining solution containing 0.292 g NaCl in 50 mL H _2_O supplemented with 15 μL dye. Gels were imaged to evaluate RNA band shifts corresponding to protein binding or displacement by suramin.

### NMR titration assays

2D ^1^H- ^15^N HSQC spectra were acquired using a Bruker Avance III 850 MHz NMR spectrometer (Bruker BioSpin GmbH, Karlsruhe, Germany) equipped with a triple-resonance ^1^H/ ^13^C/ ^15^N TCI cryoprobe (Bruker AG, Fällanden, Switzerland). The ^15^N-labelled N-CTD protein was dissolved in buffer B supplemented with 10% (v/v) D _2_O to a final volume of 500 μL. Spectra were recorded using Bruker TopSpin 3.2 software and processed with NMRPipe, whereas peak analysis and visualization were performed via NMRFAM-SPARKY [ 16, 17].

Titration experiments were performed by adding RNA or suramin to 200 μM ^15^N-labelled N-CTD at a 1:1 molar ratio. The ^1^H- ^15^N HSQC spectra captured backbone amide signals that are highly sensitive to environmental changes in protein structure. Chemical shift perturbation (CSP) and intensity perturbation index (IPI) values were calculated for each resonance peak after ligand addition via the following empirical formulas [18].

$$\begin{array}{cr} \text{∆δ =}\sqrt{\frac{\text{1}}{\text{2}}[{\left({∆\text{δ}}_{\text{H}}\right)}^{\text{2}}\text{+0}\text{.14}{\left({∆\text{δ}}_{\text{N}}\right)}^{\text{2}}]} & \text{(1)} \end{array}$$

where Δδ _H_ and Δδ _N_ represent the N-CTD chemical shift changes in the ^1^H and ^15^N nuclei, respectively, during the titration process.

$$\begin{array}{cr} \text{IPI = 1}-\frac{{\text{H}}_{\text{ligand}}}{{\text{H}}_{\text{free}}} & \text{(2)} \end{array}$$

where H _free_ and H _ligand_ represent the peak heights before and after the addition of the ligand, respectively.

### Molecular docking

Molecular docking of SARS-CoV-2 N-CTD with suramin was performed using the HADDOCK webserver ( https://wenmr.science.uu.nl/haddock2.4/) [ 19, 20]. The three-dimensional (3D) structure of N-CTD was obtained from the Protein Data Bank (PDB ID: 6YUN), whereas the suramin structure was obtained from the DrugBank database ( https://go.drugbank.com/drugs/DB04786). The docking sites were guided by interaction sites identified in NMR titration experiments. We set the number of rigid-body structures for docking to 10,000, all the numbers of semi-flexible refinements, final refinements and analyses to 1000. The lowest-energy conformation model with the highest score was analyzed by using LigPlot [ 21, 22].

Additionally, the binding modes of both RNA and suramin to the full-length SARS-CoV-2 nucleocapsid (N) protein were predicted using AlphaFold3 ( https://alphafoldserver.com/ ) [23]. The amino acid sequence of the N protein (UniProt ID: P0DTC9) and the RNA sequence (5′-AUAUGGAAGAGCCCUA-3′) were submitted to AlphaFold3 to predict the structure of the N-RNA complex. The full-length N protein structure predicted by AlphaFold3 was then used, along with the suramin structure, as input for HADDOCK to model the N-suramin interaction. The structural representations were prepared with PyMOL.

### NMR relaxation measurement

Backbone relaxation experiments were performed at 25°C on a Bruker Avance III 850 MHz NMR spectrometer using 200 μM ^15^N-labelled N-CTD. Longitudinal (R _1_) and transverse (R _2_) relaxation rates and ^1^H- ^15^N heteronuclear NOE (hNOE) values were measured to characterize the dynamic properties of the protein. For R _1_ measurements, longitudinal relaxation delays (T _1_) were set to 0.01 s, 0.05 s, 0.1 s (×2), 0.2 s, 0.4 s, 0.6 s, 0.8 s (×2), 1.2 s, 1.6 s and 2.0 s. For R _2_ measurements, transverse relaxation delays (T _2_) were set at 16.32 ms, 32.64 ms (×2), 48.96 ms, 65.28 ms, 81.60 ms, 97.92 ms, 114.24 ms, 130.56 ms (×2), 146.88 ms and 163.20 ms. The hNOE experiment consisted of two sets: a control (nonsaturated) group with a 5-s relaxation delay and a presaturated group with a 3-s saturation followed by a 2-s relaxation delay. Each experiment recorded two consecutive parallel trials to allow for accurate error estimation. The relaxation rates (R _1_ and R _2_) were calculated by fitting the peak intensities to a mono-exponential decay function using NMRFAM-SPARKY software. The hNOE values were determined by comparing the intensities of the corresponding amide peaks between the presaturated and control spectra.

### Model-free analysis

Backbone dynamics parameters, including the generalized order parameter (S²) and internal motion correlation time (τ _e_), were calculated using the FAST-Modelfree program (version 1.3; Loria Lab, New Haven, USA) [24]. A first estimation of the rotational diffusion tensor for the N-CTD was performed using the Tensor 2 software [25]. The diffusion tensor and the 3D structure of the N-CTD (PDB ID: 6YUN) were provided as inputs for the FAST model-free analysis. The tensor was iteratively refined through multiple rounds of optimization until convergence was achieved. The final values for S² and τ _e_ were extracted, providing quantitative insight into residue-specific internal motions over picoseconds and nanoseconds.

## Results and Discussion

### SARS-CoV-2 N-CTD exhibits greater binding affinity for Surmain than does RNA

**Figure 1:**
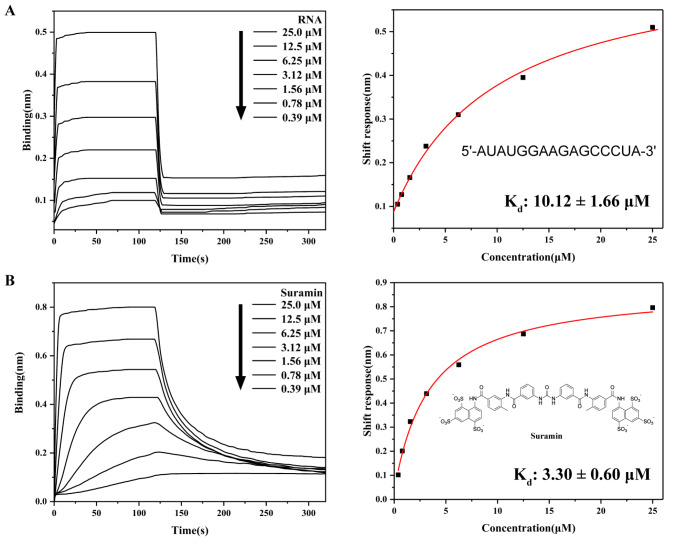
Figure 1 BLI analysis of ligand binding to SARS-CoV-2 N-CTD (A) Binding kinetics of N-CTD with RNA. (B) Binding kinetics of N-CTD with suramin.

Approximately 95% pure SARS-CoV-2 N-CTD protein was prepared by Ni-NTA affinity purification ( Supplementary Figure S1). N-CTD exists in a dimeric form whether it binds to suramin or not ( Supplementary Figure S2). Furthermore, the binding affinities of N-CTD to RNA and suramin were evaluated using biolayer interferometry (BLI). As shown in Figure 1, RNA exhibited faster association and dissociation kinetics with N-CTD ( Figure 1A) than suramin ( Figure 1B). Despite these kinetic differences, both interactions occurred in the micromolar (μM) affinity range. Notably, the dissociation constant ( *K*_d_) of RNA for N-CTD was approximately 10.12 μM, almost twice that of suramin ( *K*_d_, 3.30 μM). These results indicate that suramin binds to N-CTD with higher affinity than does RNA, suggesting its potential to competitively disrupt N-CTD-RNA interactions.

### Suramin disturbs the interaction between SARS-CoV-2 N-CTD and RNA

**Figure 2:**
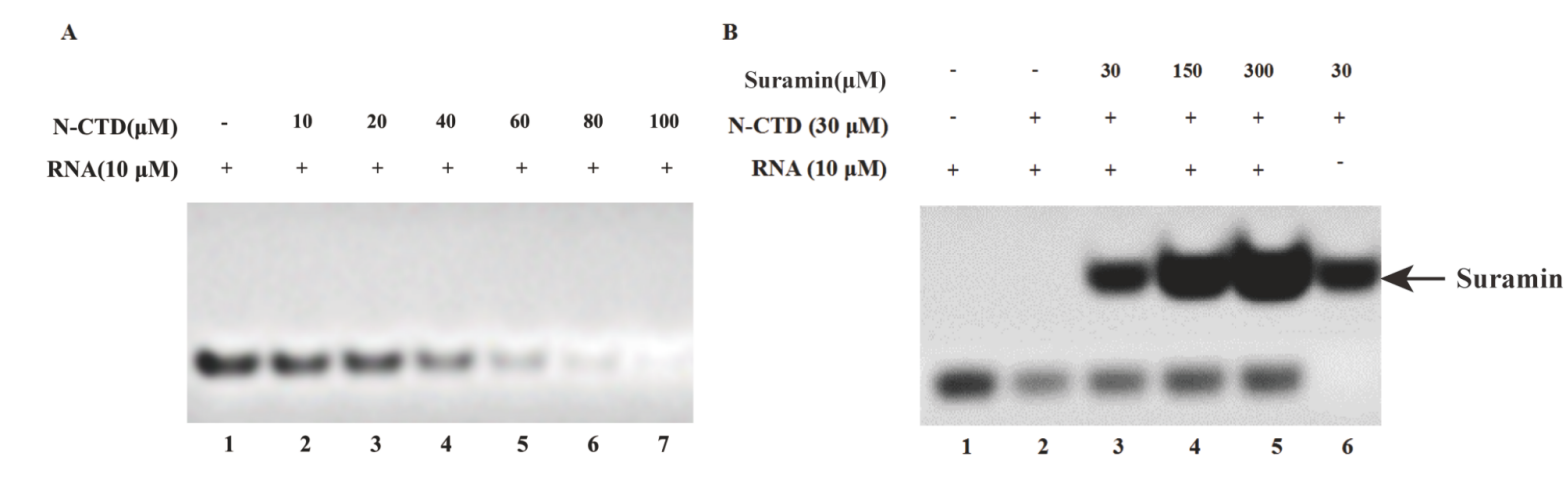
Figure 2 EMSAs evaluating the interaction between SARS-CoV-2 N-CTD and RNA (A) RNA binding by N-CTD at increasing molar ratios of 1:1, 2:1, 4:1, 6:1, 8:1 and 10:1 (lanes 2–7). Symbols (+) and (–) denote the presence or absence of components in the reaction mixture, respectively. (B) Suramin-mediated disruption of the N-CTD-RNA complex at molar ratios of N-CTD:RNA:suramin = 3:1:3, 3:1:15 and 3:1:30 (lanes 3–5).

To further evaluate the ability of suramin to disrupt the N-CTD-RNA interaction, electrophoretic mobility shift assays (EMSAs) were performed. Owing to the high positive charge of N-CTD, the protein-RNA complex migrated in the opposite direction of electrophoresis, rendering the complex band undetectable on the gel. However, the intensity of the free RNA band progressively decreased with increasing concentrations of N-CTD, indicating efficient sequestration of RNA by N-CTD ( Figure 2A). Conversely, the band corresponding to free RNA gradually intensified with increasing suramin concentration ( Figure 2B), reflecting RNA displacement from the N-CTD complex. Taken together, these results confirm that suramin effectively disrupts the N-CTD-RNA interaction *in vitro* .

### Suramin and RNA share overlapping binding sites on SARS-CoV-2 N-CTD

**Figure 3:**
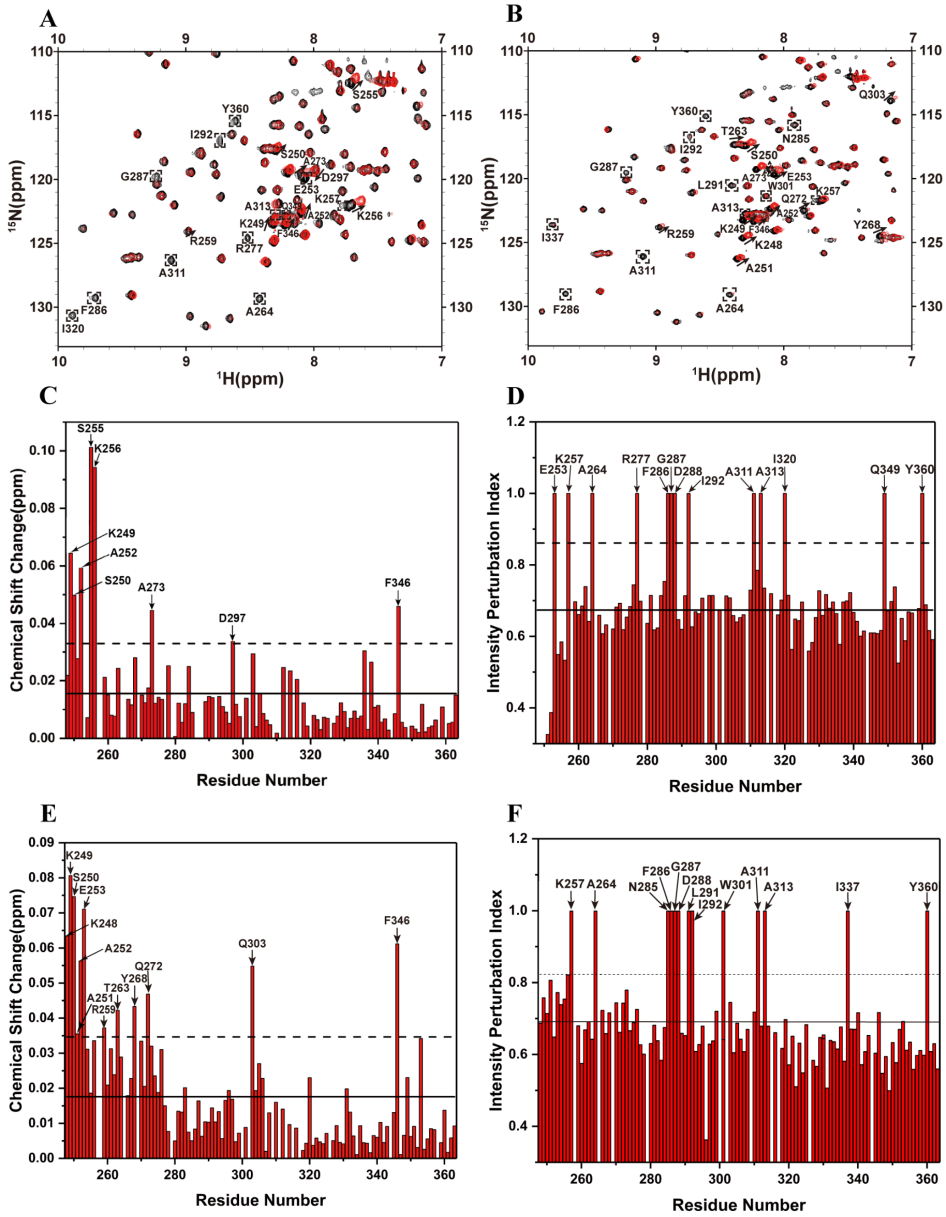
Figure 3 Identification of ligand-binding sites on SARS-CoV-2 N-CTD (A,B) 2D 1H- 15N HSQC spectra of 15N-labelled N-CTD titrated with RNA (A) and suramin (B) at a molecular ratio of 1:1, highlighting chemical shift perturbations upon ligand binding: black:N-CTD; red:N-CTD bound with RNA or suramin. (C,D) CSP and IPI plots for N-CTD titrated with RNA. (E,F) CSP and IPI plots for N-CTD titrated with suramin. The solid lines denote the mean values; the dashed lines indicate the threshold defined by the mean plus one standard deviation (SD).

To elucidate the binding interfaces of RNA and suramin on SARS-CoV-2 N-CTD, two-dimensional (2D) ^1^H- ^15^N HSQC spectra were acquired for ^15^N-labelled N-CTD titrated with each ligand. Changes in chemical shifts and signal intensities during titration allowed the identification of residues involved in ligand binding. Upon RNA addition, several N-CTD peaks exhibited significant chemical shift perturbations, broadening, or disappearance ( Figure 3A), indicating moderate binding affinity in the micromolar range. A similar spectral pattern was observed upon titration with suramin ( Figure 3B), suggesting comparable binding behavior. To identify the interacting residues, we analyzed the CSP and IPI values, selecting residues with values greater than the mean plus one standard deviation ( Figure 3C–F), on the basis of the resonance assignment of N-CTD (BMRB: 50518).

**Figure 4:**
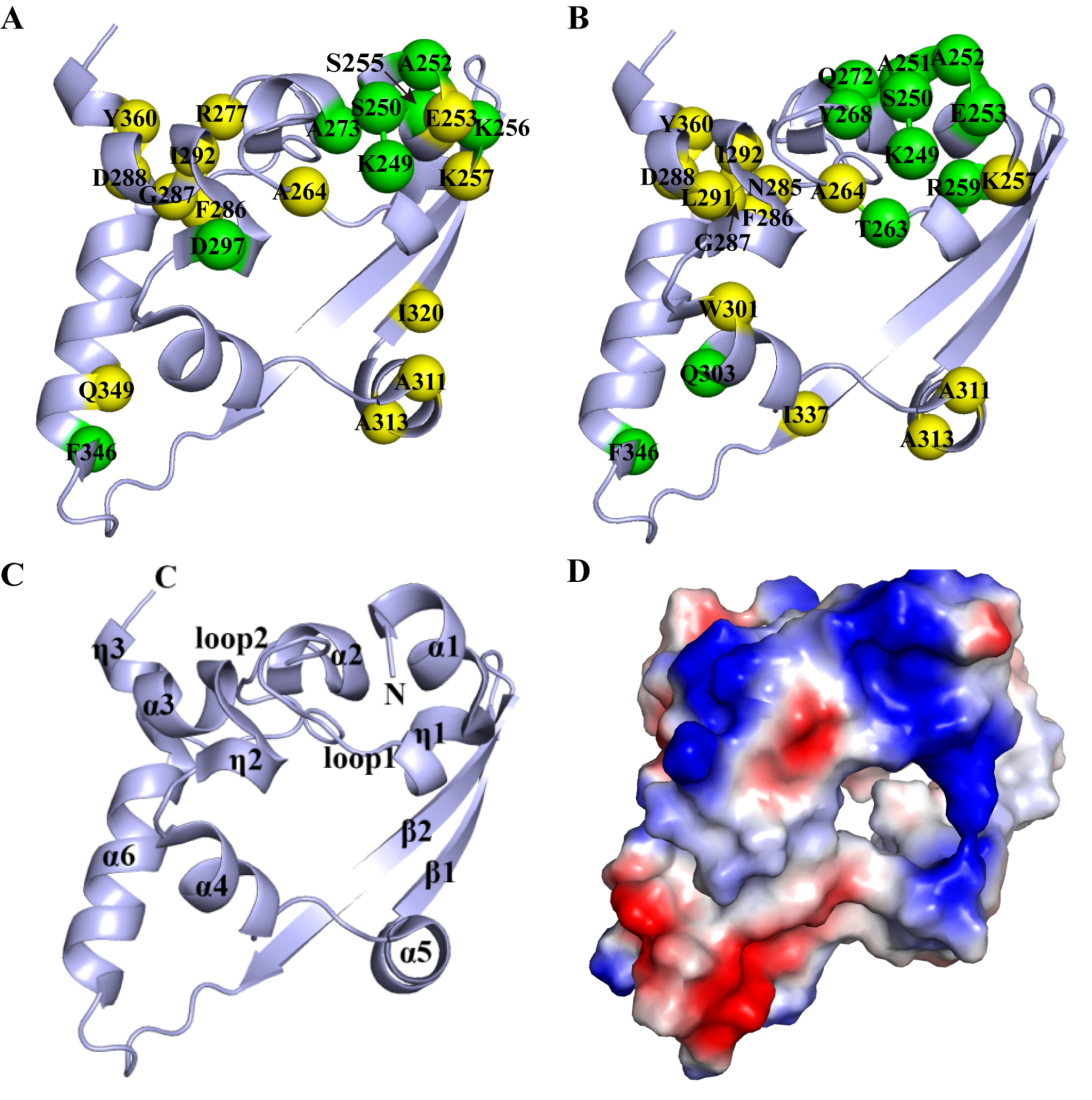
Figure 4 Structural visualization of ligand-binding sites on the SARS-CoV-2 N-CTD (A) Mapping of residues perturbed by RNA binding to the N-CTD structure. (B) Mapping of residues perturbed by suramin binding to the N-CTD structure. Residues with CSP values exceeding the mean plus one SD are shown in green; residues with elevated IPI values above the same threshold are shown in yellow. (C) Ribbon diagram and topological representation of the N-CTD structure. (D) Electrostatic surface potential of N-CTD: blue indicates positively charged regions, red indicates negatively charged regions, and white indicates neutral areas.

These perturbed residues were then mapped onto the three-dimensional structure of N-CTD (PDB ID: 6YUN). The RNA-interacting residues were predominantly located in α1 (K249, S250, A252, E253, S255, K256, K257), loop1 (A264), α2 (A273), loop2 (R277, F286, G287, D288), α3 (I292), η2 (D297), α5 (A311, A313), β1 (I320), α6 (F346, Q349) and η3 (Y360) ( Figure 4A). Suramin-binding residues included α1 (K249, S250, A251, A252, E253, K257), η1 (R259), loop1 (T263, A264, Y268), α2 (Q272), loop2 (N285, F286, G287, D288), α3 (L291, I292), α4 (W301, Q303), α5 (A311, A313), β2 (I337), α6 (F346) and η3 (Y360) ( Figure 4B). Comparative analysis revealed substantial overlap in the binding sites for RNA and suramin, particularly within the positively charged α1-η1 helix region ( Figure 4C,D). This strongly suggests that suramin competitively occupies the RNA-binding interface of N-CTD, thereby disrupting its association with viral RNA.

### Structural model of the N-CTD-suramin complex

**Figure 5:**
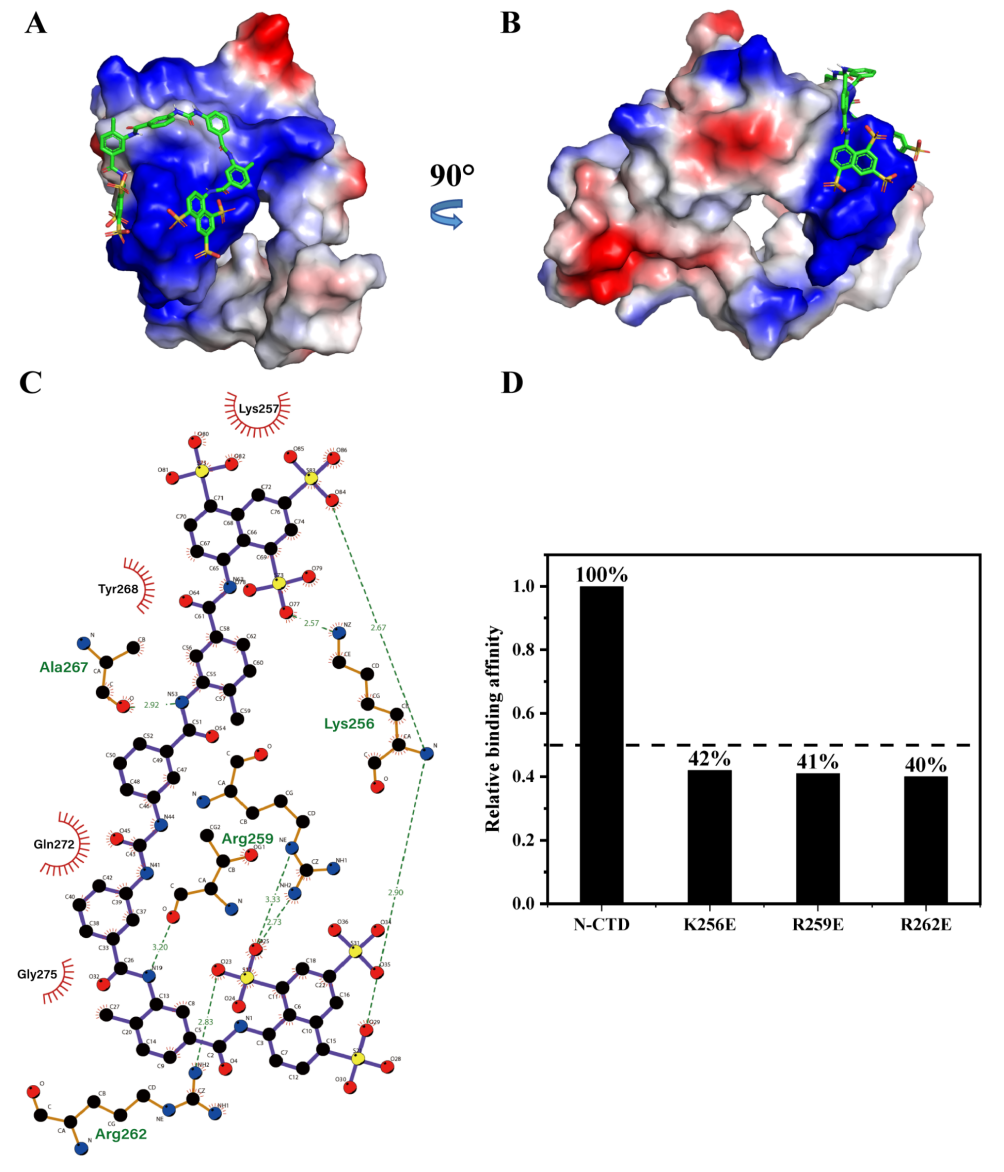
Figure 5 Key residues involved in the interaction between SARS-CoV-2 N-CTD and suramin (A,B) Structural model of the SARS-CoV-2 N-CTD-suramin complex. The 3D structure of suramin is shown as rainbow sticks. (C) Schematic illustration of the molecular interactions between N-CTD and suramin generated using LigPlot by docking analysis. (D) Comparison of the binding affinities of WT N-CTD and single-point mutants (K256E, R259E, and R262E) to suramin, as measured by BLI. The wild type binding affinity was normalized to 100%; the dashed line indicates 50% relative affinity.

To elucidate the structural basis of suramin binding to SARS-CoV-2 N-CTD, a molecular docking model was constructed on the basis of NMR titration data. As shown in Figure 5A,B, suramin is accommodated primarily within the positively charged α1-η1 helical region of N-CTD. The key interacting residues include K256, R259, R262, and A267 ( Figure 5C). Specifically, the backbone amino group of K256 forms two hydrogen bonds with sulfonic acid moieties at either end of the suramin molecule, whereas its side-chain amino group forms a salt bridge with an additional sulfonic acid group. R259 contributes two more salt bridges via its guanidinium side chain, and R262 participates in another salt bridge interaction. Moreover, the carbonyl group of A267 forms a hydrogen bond with the amino group on the extended chain of suramin. These results highlight the importance of electrostatic interactions in stabilizing the N-CTD-suramin complex.

To validate the functional significance of these positively charged residues, we generated three single-point N-CTD mutants (K256E, R259E, and R262E) and measured their binding affinities to suramin via BLI ( Figure 5D). Each mutant exhibited an approximately 60% decrease in binding affinity relative to that of the wild type (WT), underscoring the essential contribution of residues K256, R259 and R262 to suramin recognition and complex stabilization.

**Figure 6:**
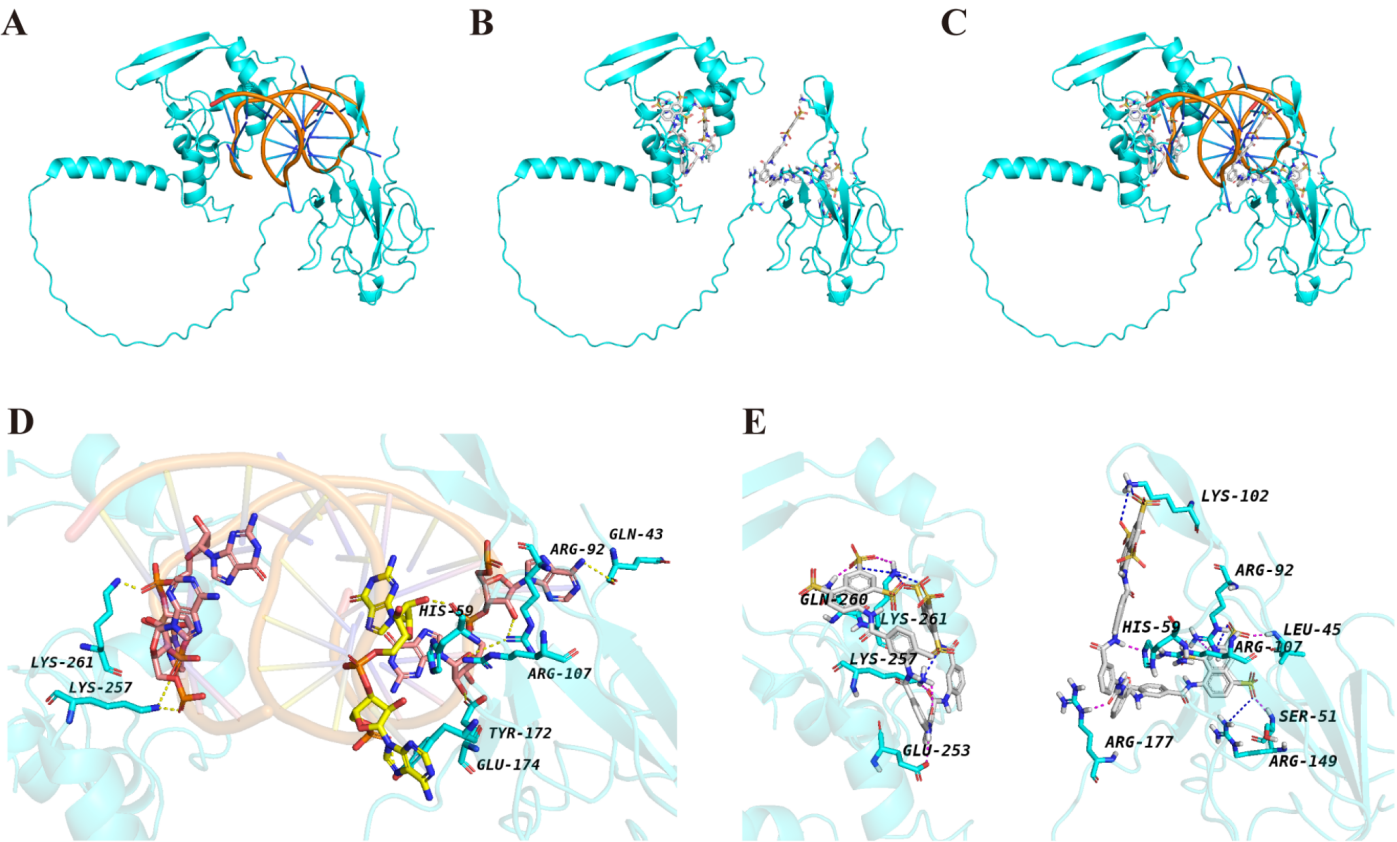
Figure 6 Predicted binding modes of RNA and suramin with the full-length SARS-CoV-2 N protein using AlphaFold 3 (A–C) Overall structural models of the N protein in complex with RNA (A), suramin (B), both RNA and suramin (C). (D,E) Close-up views of the binding interfaces: key residues involved in interactions with RNA (D) and suramin (E) are highlighted.

We further predicted the N-RNA and N-suramin complex structures via AlphaFold3 ( https://alphafoldserver.com/). As shown in Figure 6, both RNA and suramin occupy the full-length N protein in the overlapping region, which is consistent with our binding areas in the NTD and CTD determined via NMR. ITC experiments also revealed that the N protein has multiple binding sites with RNA and suramin ( Supplementary Figure S3). In particular, suramin interacts with residues in both the NTD and CTD domains that we previously identified as binding sites via NMR. Similarly, RNA binds across the same positively charged surfaces, highlighting the competitive relationship between RNA and suramin. EMSA experiments also demonstrated that suramin can dissociate RNA from the N protein ( Supplementary Figure S4).

### Dynamic properties of SARS-CoV-2 N-CTD

Protein dynamics describe temporal fluctuations in conformation and serve as an important complement to static structural information. To gain mechanistic insight into the functional flexibility of SARS-CoV-2 N-CTD, we performed NMR relaxation experiments in which the backbone amide groups were targeted. Specifically, we measured the longitudinal relaxation rate (R _1_), transverse relaxation rate (R _2_), and ^1^H- ^15^N heteronuclear steady-state nuclear Overhauser effect (hNOE) for 102 well-resolved resonance peaks.

**Figure 7:**
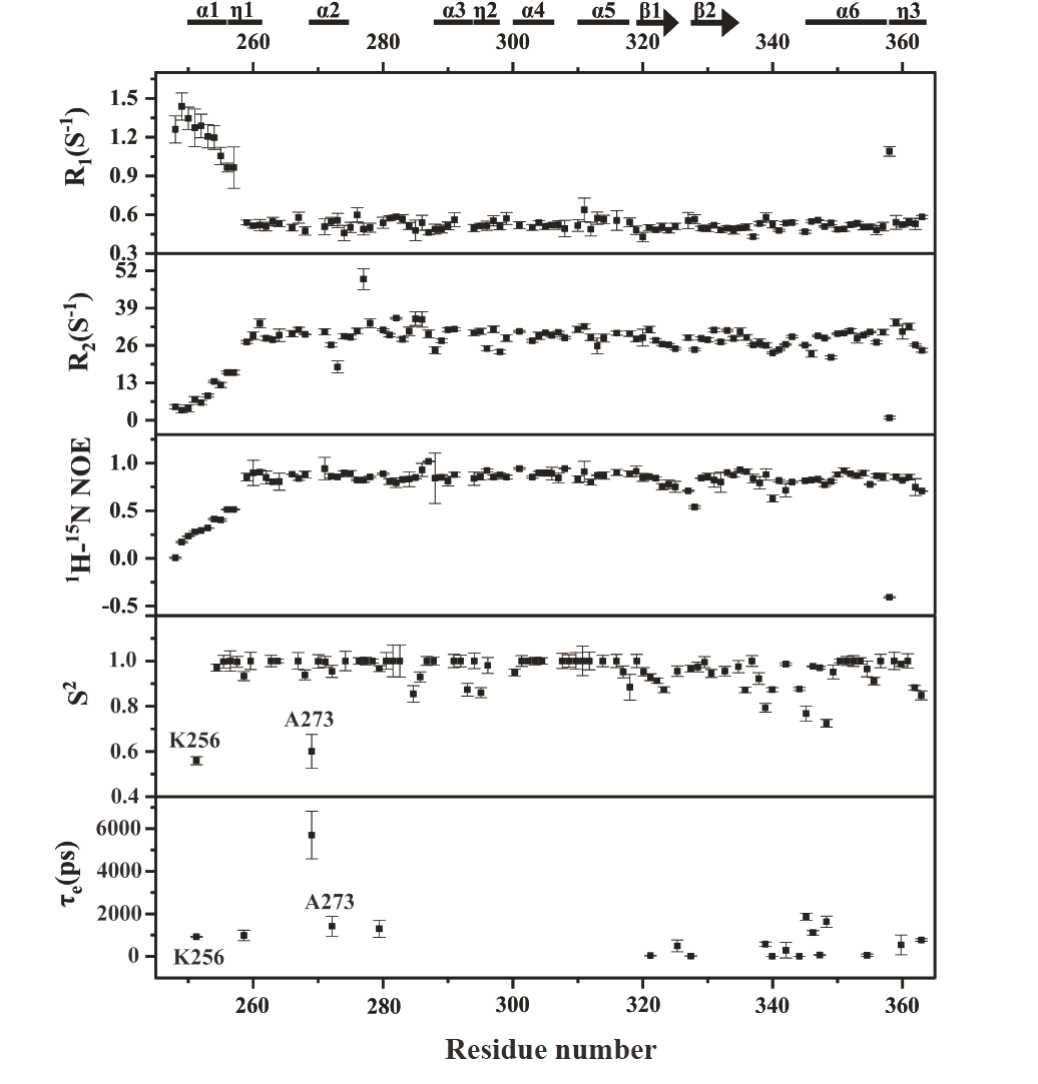
Figure 7 NMR relaxation analysis of SARS-CoV-2 N-CTD Plots of R1, R 2, 1H-15N hNOE, S² and τe values as a function of residue number, revealing residue-specific backbone dynamics across the N-CTD structure.

The R _1_ values of N-CTD ranged from 0.4 to 1.5 s ^–1^, with a mean of 0.59 s ^–1^. As shown in Figure 7, residues within the N-terminal α1-η1 helix (248–262 aa) presented elevated R _1_ values relative to the average, indicating rapid internal motion on the picosecond-to-nanosecond (ps-ns) timescale and structural flexibility in this region. The R _2_ values consistently ranged from 0 to 49 s ^–1^, with an average of 26.9 s ^–1^. Notably, residues in the α1-η1 segment presented lower than average R _2_ values, further supporting their dynamic nature.

As shown in Figure 4, the α1-η1 helix is a key ligand-binding interface on the N-CTD. Its increased flexibility, reflected in both the R _1_ and R _2_ profiles, suggests a favourable dynamic environment for ligand accommodation. In addition, the hNOE value for this region averaged 0.78, which was lower than the overall average N-CTD value, confirming its conformational flexibility. Taken together, these data demonstrate that rapid motion within the α1-η1 helix facilitates ligand binding and contributes to the functional adaptability of the N-CTD.

To characterize the internal dynamics of the N-CTD further, we used the FAST-Modelfree program to analyze the NMR relaxation data (R _1_, R _2_ and hNOE) and derive dynamic parameters, including the generalized order parameter (S ^2^) and the internal motion correlation time (τ _e_). As shown in Figure 7, the average S ^2^ value was 0.95, indicating that the N-CTD structure is largely rigid. Notably, residues K256 and A273 presented significantly reduced S ^2^ values coupled with measurable τ _e_ values, suggesting increased flexibility and rapid internal motion on the ps to ns timescale, which is likely relevant to their role in ligand binding.

The highly conserved nucleocapsid (N) protein is a promising drug target for developing antivirals against SARS-CoV-2. In this study, we found that suramin can bind to the C-terminal domain of the SARS-CoV-2 N protein (SARS-CoV-2 N-CTD) with a greater affinity than can RNA and disrupt the N-CTD-RNA interaction. Suramin is accommodated primarily within the positively charged α1-η1 helical region of the N-CTD, with residues K256, R259 and R262 being critical for ligand recognition. NMR dynamics analysis revealed that the high flexibility on the picosecond to nanosecond timescale within the α1-η1 helix facilitates suramin engagement and contributes to the functional adaptability of the N-CTD. Our study provides the structural basis by which suramin disturbs the N-CTD-RNA interaction, which is beneficial for the design of targeted antivirals against SARS-CoV-2.

## Supplementary Data

Supplementary data are available at *Acta Biochimica et Biophysica Sinica* online.
